# Are YAP and TAZ valid prognostic signatures for NSCLC patients?

**DOI:** 10.1111/jcmm.17992

**Published:** 2023-10-11

**Authors:** Antonios N. Gargalionis, Kostas A. Papavassiliou, Athanasios G. Papavassiliou

**Affiliations:** ^1^ Department of Biopathology, ‘Eginition’ Hospital, Medical School National and Kapodistrian University of Athens Athens Greece; ^2^ First University Department of Respiratory Medicine ‘Sotiria’ Hospital, Medical School, National and Kapodistrian University of Athens Athens Greece; ^3^ Department of Biological Chemistry, Medical School National and Kapodistrian University of Athens Athens Greece

To the Editor,

It is well established that cancer cell biomechanics represents an important factor engaged in non‐small cell lung cancer (NSCLC) development and progression. Mechanosensor proteins mediate an interplay between cancer cells, alterations of the extracellular matrix (ECM) stiffness occurring during tumour expansion and cells from the tumour microenvironment (TME). These interactions foster oncogenic signalling, which promotes NSCLC cell proliferation, angiogenesis, invasion, metastasis and resistance to anticancer drugs. Recent studies led to the discovery of biomarkers and specific molecular targeting against mechanobiological aspects of solid tumours to tailor personalized treatments.^1^ Yes‐associated protein (YAP) and transcriptional coactivator with PDZ‐binding motif (TAZ), the final effectors of the Hippo signalling pathway, are core mechanoresponsive transcriptional regulators that integrate molecular circuits of mechanotransduction. Distortion of such circuits has been implicated in NSCLC initiation, progression, metastasis, stemness and resistance to therapy.^2^ In this regard, experimental data suggest that YAP and TAZ could represent reliable biomarkers in NSCLC.

Collagen fibres are abundant components of ECM in lung cancer tissues. They are associated with augmented ECM rigidity and dysregulated mechanosignaling. Quantitative image analysis of collagen fibres has revealed that the YAP/TAZ pathway transcriptionally controls a distinct subset of genes displaying increased expression in lung carcinomas. This gene upregulation is correlated with ECM stiffness in NSCLC but not in small cell lung carcinomas (SCLC), implying that the tumour‐promoting effects of ECM stiffening may be applicable specifically to lung adenocarcinomas.^3^ Lung carcinomas with increased collagen fibre density also exhibit elevated expression of the immune checkpoint molecule, programmed death ligand 1 (PD‐L1).^3^ YAP and TAZ affect tumour immunity by modulating the expression of PD‐L1 through tethering to the *PD‐L1* promoter and via the TEA domain family member (TEAD) group of transcription factors, respectively. YAP and TAZ stimulate immunosuppression in NSCLC; therefore, they can be used in combination regimens with immune checkpoint inhibitors but also as predictive biomarkers in the context of immune checkpoint inhibition.^4^

A collection of studies has evaluated the association of YAP/TAZ expression in NSCLC with clinical, pathological and survival parameters. In a comprehensive review and meta‐analysis, the expression levels of YAP and TAZ and their role as prognostic markers were assessed in a total of 1977 patients across five studies with lung cancers. High YAP expression correlates with decreased overall survival (OS), especially in the Asian population.^5^, ^6^, ^7^, ^8^, ^9^, ^10^, ^11^ Evaluation of TAZ expression in association with OS of patients with NSCLC showed that elevated expression of TAZ is correlated with decreased OS in the overall population and, particularly, in the Caucasian and the Asian populations.^12^, ^13^, ^14^ TAZ expression has been also linked specifically to lung adenocarcinomas, poor differentiation, advanced tumour, node and metastasis (TNM) stage and poor prognosis of OS (*n* = 181).^14^ The meta‐analysis of NSCLC patients, including 414 cases from six studies, also indicates that elevated YAP1 expression is associated with decreased OS. Furthermore, YAP1 high expression correlates with poor progression‐free survival (PFS). When the cases were stratified according to population, studies that were conducted in Asia, Korea and China revealed a significant association of YAP1 high expression with OS and PFS. Nevertheless, this was not the case with studies that were conducted in Spain.^11^ Corroborating findings from the evaluation of YAP1 mRNA expression in various tumour types present downregulated YAP1 expression in lung adenocarcinoma and lung squamous cell carcinoma.^15^ The studies showing YAP1 downregulation and nonsignificant survival associations only assessed mRNA expression, thus suggesting that protein detection and evaluation of protein expression levels are essential to determine YAP1 prognostic significance. When cases were stratified according to TNM stage (*n* = 155), high YAP1 expression was only associated with PFS.

YAP/TAZ demonstrate distinct roles in NSCLC‐specific subsets. High YAP1 nuclear expression is associated with poor OS and PFS in NSCLC patients who have been treated with tyrosine kinase inhibitors (TKIs) against epidermal growth factor receptor (EGFR), compared with patients that were not treated with EGFR‐TKIs. EGFR‐TKI‐treated patients have a higher mutation rate of EGFR, where nuclear YAP1 activity is associated with resistance to EGFR‐TKIs.^11^ Accordingly, YAP1 seems promising for application not only as a prognostic marker for EGFR‐TKI therapy but also in combinatorial EGFR‐TKI/YAP1 inhibition treatments. TAZ, however, enhances angiogenesis and promotes tumour growth in EGFR wild‐type NSCLC cells and xenografts, respectively. Mechanistically, TAZ upregulates the EGFR ligand amphiregulin, potentiates EGFR signalling and sensitizes EGFR wild‐type cells to the EGFR inhibitor gefitinib. This observation indicates that TAZ can be employed as a biomarker regarding EGFR genotype for efficient EGFR‐TKI therapy.^16^

The above indicatory data pose that YAP and TAZ can be effective prognostic biomarkers in NSCLC. Moreover, they are associated with specific NSCLC subgroups, such as patients with lung adenocarcinoma, EGFR‐TKI‐treated patients and patients with advanced TNM stage. Future investigations should aim to map the complex network underpinning the interactions between NSCLC, ECM and TME cells. Decoding the YAP/TAZ‐mediated molecular mechanisms in various clinical settings can provide novel biomarkers and therapeutic targets to overcome drug resistance in NSCLC.

## AUTHOR CONTRIBUTIONS

**Antonios N. Gargalionis:** Conceptualization (equal); data curation (equal); writing – original draft (lead). **Kostas A. Papavassiliou:** Conceptualization (equal); data curation (equal); writing – original draft (equal). **Athanasios G. Papavassiliou:** Conceptualization (lead); data curation (lead); supervision (lead); writing – review and editing (lead).

## FUNDING INFORMATION

None.

## CONFLICT OF INTEREST STATEMENT

The authors declare no competing financial interests.

## Data Availability

Data sharing is not applicable—no new data generated.

## References

[1] Papavassiliou KA , Basdra EK , Papavassiliou AG . The emerging promise of tumour mechanobiology in cancer treatment. Eur J Cancer. 2023;190:112938. 37390803 10.1016/j.ejca.2023.112938

[2] Lo Sardo F , Strano S , Blandino G . YAP and TAZ in lung cancer: oncogenic role and clinical targeting. Cancers (Basel). 2018;10(5):137. 29734788 10.3390/cancers10050137 PMC5977110

[3] Almici E , Arshakyan M , Carrasco JL , et al. Quantitative image analysis of Fibrillar collagens reveals novel diagnostic and prognostic biomarkers and histotype‐dependent aberrant mechanobiology in lung cancer. Mod Pathol. 2023;36(7):100155. 36918057 10.1016/j.modpat.2023.100155

[4] Papavassiliou KA , Marinos G , Papavassiliou AG . Targeting YAP/TAZ in combination with PD‐L1 immune checkpoint inhibitors in non‐small cell lung cancer (NSCLC). Cell. 2023;12(6):871. 10.3390/cells12060871 PMC10047112 36980211

[5] Chaib I , Karachaliou N , Pilotto S , et al. Co‐activation of STAT3 and YES‐associated protein 1 (YAP1) pathway in EGFR‐mutant NSCLC. J Natl Cancer Inst. 2017;109(9):djx014. 28376152 10.1093/jnci/djx014 PMC5409000

[6] Hong SA , Jang SH , Oh MH , Kim SJ , Kang JH , Hong SH . Overexpression of YAP1 in EGFR mutant lung adenocarcinoma prior to tyrosine kinase inhibitor therapy is associated with poor survival. Pathol Res Pract. 2018;214(3):335‐342. 29487002 10.1016/j.prp.2018.01.010

[7] Jiang Y , Xie WJ , Chen RW , et al. The hippo signaling Core components YAP and TAZ as new prognostic factors in lung cancer. Front Surg. 2022;9:813123. 35388363 10.3389/fsurg.2022.813123 PMC8977465

[8] Karachaliou N , Gonzalez‐Cao M , Crespo G , et al. Interferon gamma, an important marker of response to immune checkpoint blockade in non‐small cell lung cancer and melanoma patients. Ther Adv Med Oncol. 2018;10:1758834017749748. 29383037 10.1177/1758834017749748 PMC5784541

[9] Kim MH , Kim YK , Shin DH , et al. Yes associated protein is a poor prognostic factor in well‐differentiated lung adenocarcinoma. Int J Clin Exp Pathol. 2015;8(12):15933‐15939. 26884866 PMC4730079

[10] Wang Y , Dong Q , Zhang Q , Li Z , Wang E , Qiu X . Overexpression of yes‐associated protein contributes to progression and poor prognosis of non‐small‐cell lung cancer. Cancer Sci. 2010;101(5):1279‐1285. 20219076 10.1111/j.1349-7006.2010.01511.x PMC11158334

[11] Zhu L , Ma G , Liu J , et al. Prognostic significance of nuclear yes‐associated protein 1 in patients with nonsmall cell lung cancer: a systematic review and meta‐analysis. Medicine (Baltimore). 2019;98(16):e15069. 31008931 10.1097/MD.0000000000015069 PMC6494286

[12] Malik SA , Khan MS , Dar M , Hussain MU , Mudassar S . TAZ is an independent prognostic factor in non‐small cell lung carcinoma: elucidation at protein level. Cancer Biomark. 2017;18(4):389‐395. 28128737 10.3233/CBM-160263 PMC13020624

[13] Noguchi S , Saito A , Horie M , et al. An integrative analysis of the tumorigenic role of TAZ in human non‐small cell lung cancer. Clin Cancer Res. 2014;20(17):4660‐4672. 24951773 10.1158/1078-0432.CCR-13-3328

[14] Xie M , Zhang L , He CS , et al. Prognostic significance of TAZ expression in resected non‐small cell lung cancer. J Thorac Oncol. 2012;7(5):799‐807. 22481233 10.1097/JTO.0b013e318248240b

[15] Hu X , Zhang Y , Yu H , et al. The role of YAP1 in survival prediction, immune modulation, and drug response: a pan‐cancer perspective. Front Immunol. 2022;13:1012173. 36479120 10.3389/fimmu.2022.1012173 PMC9719955

[16] Yuan W , Xu W , Li Y , et al. TAZ sensitizes EGFR wild‐type non‐small‐cell lung cancer to gefitinib by promoting amphiregulin transcription. Cell Death Dis. 2019;10(4):283. 30911072 10.1038/s41419-019-1519-z PMC6433914

